# In This Issue

**DOI:** 10.1002/cfg.283

**Published:** 2003-04


**Comparative and Functional Genomics**
*Comp Funct Genom* 2003; **4**: 169.
Published online 1 April 2003 in Wiley InterScience (www.interscience.wiley.com). **DOI:** 10.1002/cfg.283

### In this issue

### Gene selection in arthritis classification with microarray expression profiles

Naijun Sha *et al.* describe a new method for the identification of multiple gene predictors of disease class, which they apply to the classification of rheumatoid arthritis and osteoarthritis samples.

### Featured organism: Teleostei genomics

Melody Clark reports on the progress made and resources available for genomics of bony fish, from models such as zebrafish and *Fugu*, to important aquaculture species such as salmon and catfish.

### Featured organism: nematode genomics

William Gregory and John Parkinson bring us up-to-date on nematode genomics, including what we know about the *Caenorhabditis elegans* genome (whose sequence was recently fully completed), details on studies of related nematodes and nematode functional genomics studies.

### Meeting review: HUPO Protein Standards Initiative

Sandra Orchard and colleagues report on the second meeting of the HUPO Protein Standards Initiative, which included the presentation of a draft XML model for protein–protein Interactions (PPI) data, a draft controlled vocabulary for PPI methods, and further discussions on standards and an XML model for mass spectrometry data.

### A special section of selected reviews from the Plant and Animal Genomes XI (PAGXI) meeting

David Galbraith discusses the use of microarrays, in combination with a range of techniques, for the study of cell type-specific gene expression.

Stan Wullschleger and Stephen DiFazio discuss the potential of microarrays for the study of plant physiology, highlighting existing projects that were presented at PAGXI.

Barry Rolfe *et al.* discuss the potential for proteomics to aid studies of plant–microbe interactions, using their own work on legume–symbiont interactions as an example.

Christophe Plomion *et al.* report from the forest tree genomics session at PAGXI, bringing us a cross-section of the activities of the tree genomics community and a taster of their plans for the future.

Eve Wurtele *et al.* present MetNet, a software tool for modelling the metabolic and regulatory networks of *Arabidopsis*.

Chunguang Du *et al.* present their maize molecular evolutionary genomic database.

Martin Gollery provides an overview of hidden Markov model (HMM) algorithms and databases, and how to develop custom HMM targets, such as his microbe-specific HMM databases.

Heiko Schoof discusses key issues and challenges in achieving database interoperability and describes the approach that MIPS plan to take for their *Arabidopsis* database, MAtDB.

Zhanjiang Liu reports on the progress made in catfish genomics, from mapping to the development of genomics resources, and discusses the prospects for the future.

Max Rothschild reports on the status of swine genomics, as the community awaits news on the NIH funding available for sequencing the pig genome.

Debora Hamernik and David Adelson report from the USDA stakeholder workshops held electronically and at PAGXI to discuss the needs of the community for animal genome bioinformatics and the role of the USDA in supporting those needs.

### Conference calendar

We present a round-up of genomics-related conferences planned for June–August 2003.

Copyright © 2003 John Wiley & Sons, Ltd.

